# Identification of Crucial Genetic Factors, Such as PPARγ, that Regulate the Pathogenesis of Fatty Liver Disease in Dairy Cows Is Imperative for the Sustainable Development of Dairy Industry

**DOI:** 10.3390/ani10040639

**Published:** 2020-04-07

**Authors:** Kerong Shi, Ranran Li, Zhongjin Xu, Qin Zhang

**Affiliations:** Key Laboratory of Animal Bioengineering and Disease Prevention, College of Animal Science and Technology, Shandong Agricultural University, Tai’an 271018, Shandong, China; liranranone@163.com (R.L.); 18206381011@139.com (Z.X.); qzhang@cau.edu.cn (Q.Z.)

**Keywords:** dairy cows, PPARγ, fatty liver, non-alcoholic fatty liver disease (NAFLD), genetic factor, dairy industry

## Abstract

**Simple Summary:**

Fatty liver disease frequently occurs in dairy cows, a typical type of non-alcoholic fatty liver disease (NAFLD), resulting in a high culling rate of dairy cows during the perinatal period because of limitations of lactation and reproduction performance of cows with subsequent complications. This has been developing into a worldwide crucial industrial problem. Studies about NAFLD have shown that PPARγ (peroxisome proliferator-activated receptor γ) participates or regulates the fat deposition in liver by affecting the biological processes of hepatic lipid metabolism, insulin resistance, gluconeogenesis, oxidative stress, and inflammation, which all contribute to fatty liver. This review mainly focuses on the understanding of molecular pathogenesis of fatty liver disease in dairy cows by taking PPARγ as an example, so as to provide important information for discovering critical therapeutic targets, such as PPARγ, for fatty liver disease, and contribute to breeding improvement of fatty liver disease-resistant dairy cattle and eventually sustainable development of dairy industry.

**Abstract:**

Frequently occurring fatty liver disease in dairy cows during the perinatal period, a typical type of non-alcoholic fatty liver disease (NAFLD), results in worldwide high culling rates of dairy cows (averagely about 25%) after calving. This has been developing into a critical industrial problem throughout the world, because the metabolic disease severely affects the welfare and economic value of dairy cows. Findings about the molecular mechanisms how the fatty liver disease develops would help scientists to discover novel therapeutic targets for NAFLD. Studies have shown that PPARγ participates or regulates the fat deposition in liver by affecting the biological processes of hepatic lipid metabolism, insulin resistance, gluconeogenesis, oxidative stress, endoplasmic reticulum stress and inflammation, which all contribute to fatty liver. This review mainly focuses on crucial regulatory mechanisms of PPARγ regulating lipid deposition in the liver via direct and/or indirect pathways, suggesting that PPARγ might be a potential critical therapeutic target for fatty liver disease, however, it would be of our significant interest to reveal the pathology and pathogenesis of NAFLD by using dairy cows with fatty liver as an animal model. This review will provide a molecular mechanism basis for understanding the pathogenesis of NAFLD.

## 1. Introduction

Fatty liver syndrome, a typical type of metabolic disorder, frequently occurs in populations of dairy cows in commercial farms throughout the world, which is caused by negative nutrient balance after calving. High-yielding dairy cows, especially for cows with 30 kg of daily milk yield and more, are usually inclined to develop fatty liver syndrome in the early lactation period. There are more risk factors available to developing fatty liver syndrome, such as cows with high body condition score (BCS) and/or high body fat, feed intake decreases around calving, etc.

Our investigation in the last three years indicated that 48.85% of dairy cows (*n* = 346) within 2 weeks after parturition were diagnosed with light or severe fatty liver disease by randomly selecting 710 Holstein dairy cows from four commercial farms (Zhang et al., unpublished data) [1,2]. The suspected fatty liver cows and/or normal cows were firstly distinguished by applying the previously reported model [3] using the detected values of serum biochemical traits (glucose, Glu; non-esterified fatty acid, NEFA; aspartate transaminase, AST), then representative cows with different suspected extents were biopsied for liver tissue samples for fat deposition amount assessment by oil red staining. It is estimated that 40%–60% of high-yielding dairy cows (daily milk yield > 35 kg) develop moderate to severe fatty liver disease within 2 weeks after calving [4]. Moreover, it is not uncommon for the two short weeks after parturition to account for 50% of morbidity on a dairy farm [5], which is in accordance with our results.

The perinatal disorders including fatty liver disease remain as prevalent now as they were 20 years ago [5], causing high culling rate of dairy cows in their early lactation period, which is becoming a critical concern in modern dairy industry. The average culling rate of dairy cows within 60 days of lactation (during parturition period) in populations is about 24% in USA [6,7,8], and about 27% in China [9,10]. In clinical practice, increasing blood calcium levels and using anti-inflammatory drugs after delivery in dairy cows could reduce the incidence of this disease and/or decrease the economic loss [11]. However, these strategies cannot either completely change the situation or alleviate contradictions. The incidence of fatty liver disease at the early lactation not only decreases the milk yield of the coming lactation period, but also attenuates the future milk production and reproduction performance because of subsequent health problems of dairy herds [1,2,7,12], such as ketosis, displaced abomasum, mastitis, etc. It is estimated that economic losses due to the treatment of and reduction in milk production by one dairy cow with ketosis accounts for 151–312 US dollars [11].

## 2. Ethics Approval and Consent to Participate

All the investigation and biopsy processes were carried out in accordance with guidelines issued by the Shandong Agricultural University Animal Care and Use Committee (approval number, SDAUA-2017-044). We have obtained written informed consent from the animal owner to use these animals in the study.

## 3. Progress of the Pathogenesis Mechanism of Fatty Liver Disease

The etiology of fatty liver in perinatal dairy cows primarily includes the negative nutrient balance and the accumulation of high level of free fatty acids (FFA) in serum or triglycerides (TAG) deposited in liver [1]. Liver, the central organ of organisms, regulates the metabolic balance of carbohydrate, fat, and protein in mammals [13]. After calving, the food intake of dairy cows further decreases, while lactation slowly increases. Thus, the body lactose consumption easily results in the cow to be susceptible to experiencing an insufficient sugar supply, thus promoting fat mobilization in the liver [14,15]. Additionally, energy and substance metabolism is centered in the liver. The increasing fat mobilization promotes gluconeogenesis, increases the blood sugar concentration, and alleviates the negative nutrient balance. At the same time, the enhanced fat mobilization promotes the dramatic increase of non-esterified fatty acid (NEFA) in the liver [16], which is partly re-esterified to synthesize the triglycerides (TAG), a type of very-low-density lipoprotein (VLDL), that is hardly transported out of the liver [17]. Especially for dairy cattle, TAG is excessively accumulated because of its lack for esterase, resulting in susceptibility to fatty liver disease [14,18].

As for non-alcoholic fatty liver disease (NAFLD) occurring in human beings, metabolic disorder syndromes and obesity are also usually the main causes, with increased plasma insulin and fatty acid concentration, elevated fasting aminotransferase (AST/ALT) and/or triglycerides (TAG) level, and also abnormal lipid accumulation in the liver [19,20,21]. In addition, another of the most important risk factors is histological evidence of hepatic inflammation [22] caused by acute inflammation and subacute inflammation [5]. Thus, dairy cows with fatty liver disease is a typical NAFLD animal model, good for revealing the pathology and pathogenesis of NAFLD.

In recent years, scientists have proposed a “two-hit” theory to explain the pathogenesis mechanisms of NAFLD [23,24]. (1) The “first hit” was caused by insulin resistance (IR). IR can not only strengthen lipolysis of surrounding tissues, but also causes hyperinsulinemia. The lipolysis of adipose tissue results in increased FFA and enhanced TAG synthesis in the liver. The FFA has hepatocellular toxicity, increasing the permeability of cell membrane and disrupting the mitochondrial function by inhibiting related enzymes. (2) The “second hit” was caused by the imbalance between the coexisting systems of oxidation and anti-oxidation in the liver. The increase in lipid peroxidation results in persistent reactive oxygen species (ROS) production. In addition to the pre-existing factors related to the enhanced oxygen stress, other new or additional factors can increase lipid peroxidation for a second hit to the liver, such as inflammatory cytokines, adipokines, endotoxins, and mitochondrial inactivation. The second hit will eventually lead to NASH (non-alcoholic steatohepatitis) progression beyond hepatic steatosis that promotes oxidative stress, inflammation, cell death, and fibrosis. Especially, the inflammation is positively correlated with liver injury and negatively correlated with lipolysis by inhibiting lipase activity, inhibiting the transport of lipoproteins and/or lipids and causing lipid deposition. Moreover, it also induces lipid peroxidation, IR, and cell apoptosis [25,26], aggravating the pathogenesis of NAFLD. (3) Actually, there is even a “third hit” [27], which is cell death and irreversible cell repair of hepatocytes. (4) Additionally, endoplasmic reticulum (ER) stress is another important “hit” in the pathogenesis of NAFLD. The metabolic disorders, such as obesity and diabetes, can cause ER stress, leading to the accumulation of incorrectly folded proteins (unfolded protein response, UPR) and affecting the normal physiological functions of liver cells. It is worth mentioning that ER stress can activate SREBP (sterol-regulatory element binding protein), promoting the transcription of acetyl-CoA carboxylase (ACC) and fatty acid synthase (FAS), resulting in increased synthesis of TAG and fatty acids in the liver [28,29]. Moreover, the oxidative stress (ROS producing) in liver cells can induce ER stress leading to incorrect protein fold and/or protein modification. Additionally, ER stress also can be the cause of oxidative stress. The biological process of ER stress and oxidative stress interact each other through different pathways, leading to IR and aggravating the NAFLD [30,31,32].

However, the pathogenesis of NAFLD is still unclear. It was proposed that the abnormality in lipid and lipoprotein metabolism accompanied by chronic inflammation and oxidative stress is the central pathway and/or the major risk factors involved in the pathogenesis of NAFLD [33,34]. It is generally believed that the occurrence of fatty liver is not only closely related to insulin resistance and disorder of fat metabolism, but also related to biological processes, such as glycometabolism disorder, oxidative stress, and intracellular inflammatory response [23,35]. Moreover, these processes are correlated and/or coordinated with each other and accelerate the progress of NAFLD [23,36]. There are numerous factors involved in the pathogenesis of NAFLD. For example, PPARα (peroxisome proliferator-activated receptor α) and/or PPARγ, microsomal triglyceride transport protein (MTP), apolipoprotein (apoB) play important roles in hepatic lipid metabolism, lipid transport, and secretion [35,37]; adipocytokines (Tumor necrosis factor, leptin, and adiponectin), cytokines (Interleukin-6, IL-6; glucagon-like peptide-1, GLP-1; fibroblast growth factor 19, FGF-19; fibroblast growth factor 21, FGF-21; growth-hormone-releasing hormone, GHRH; etc.), and toll-like receptors (TLRs) participate in insulin resistance (IR), oxidative stress, and inflammatory response, and mediate cell apoptosis/necrosis to promote liver fibrosis [35,38,39,40]; microRNAs (such as mir-107 and miR-103) are reported to regulate insulin resistance. Recently, RG-125 (also named AZD4076), the antagonist against microRNA-103/107, has entered the phase I clinical trial of NASH (non-alcoholic steatohepatitis) treatment [39].

Accordingly, the pathogenesis of NAFLD is usually related to abnormal hepatic lipid metabolism, gluconeogenesis, IR, oxidative stress, and inflammation [23,39,40,41,42,43]. The causal relationship and the underlying molecular mechanisms of these biological processes still remains unclear. However, the discovery of some important regulatory factors/genes that regulate all these biological processes would be helpful to reveal the molecular pathogenesis of NAFLD.

Recent studies have shown that PPARγ (peroxisome proliferator-activated receptor γ) participates or regulates lipid metabolism in the liver by affecting the biological processes of hepatic lipid metabolism, insulin resistance, gluconeogenesis, oxidative stress, and inflammation. In the following content, PPARγ will be taken as an example to elaborate the crucial regulatory role of certain critical genetic factors in the hepatic liver metabolism and therefore lipid deposition, via direct and/or indirect pathways. Rosiglitazone (RGZ) and other thiazolidinedione (TZD) synthetic ligands of PPARγ are insulin sensitizers that have been used for the treatment of type Ⅱ diabetes [44]. As for the PPARγ related substances, apigenin, a food-derived compound, were reported significantly ameliorated NAFLD and obesity-induced metabolic syndrome by acting as a PPARγ modulator through Nrf2 (nuclear factor E2-related factor 2), inhibiting the lipid metabolism and oxidative stress abnormity [45,46].

## 4. Molecular Regulatory Effects of PPARγ on the Pathogenesis of Fatty Liver Disease 

PPARs are nuclear hormone receptors that belong to the steroid hormone superfamily, playing an important role in regulating various intracellular metabolic processes [47]. PPARs comprise of three subtypes (PPARα, PPARβ, and PPARγ) that are encoded by multiple genes [48,49]. PPARα, PPARβ, and PPARγ contain 468, 441, and 479 amino acid residues, respectively [50,51]. Although they are highly homologous regions regarding their similar amino acid sequence and protein structure, PPARα, PPARβ, and PPARγ exhibit different tissue expression specificity and selectivity for different ligands [52]. PPARγ is mainly expressed in adipose and immune tissues, with low expression in the liver, kidneys, and cartilage [53]. However, PPARγ is an important participant in lipid metabolism and lipogenesis [54,55] and significantly affects hepatic glucose and lipid metabolism, adipocyte differentiation, and inflammatory responses. Studies have shown that PPARγ is activated by peroxisome proliferators as well as endogenous fatty acids and their derivatives. In addition, PPARγ participates in lipid metabolism [48] via increasing the uptake and storage of lipids as TAG in adipose tissues and highly expressing in brown fat and white fat, thereby proving its importance in adipocyte differentiation [56].

**Figure 1:**
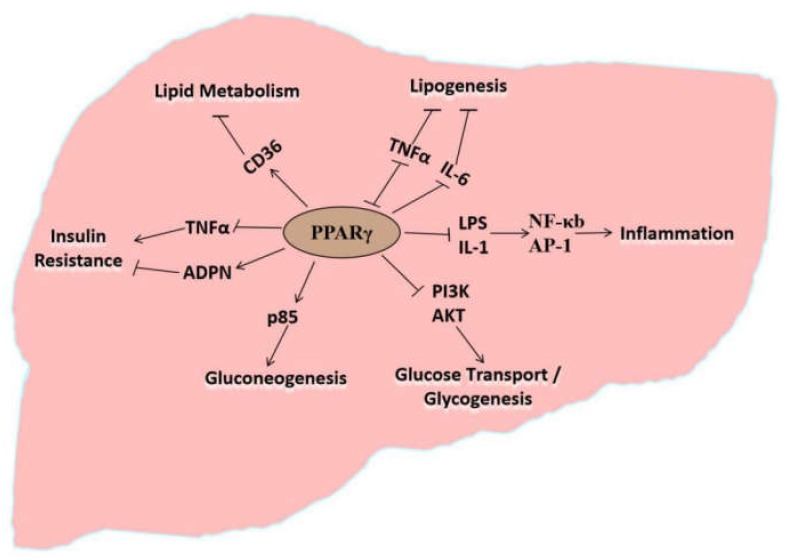
Peroxisome proliferator-activated receptor γ (PPARγ) participates in regulation of liver associated biological processes through different signaling factors and/or cytokines. Arrows indicate positive regulation and blunt arrows indicate negative regulation.

The development of fatty liver disease correlates with lipid mobilization, hepatic metabolism of free fatty acids, IR, oxidative stress, and gluconeogenesis. Studies have shown that PPARγ directly participates in lipid metabolism, thereby affecting lipid synthesis, oxidation, and transport in the liver. Moreover, PPARγ regulates the metabolic processes, such as insulin resistance (IR), inflammation, and gluconeogenesis, indirectly affecting lipid metabolism in the liver (Appendix, Table A1). As shown in Figure 1, PPARγ regulates different pathways through different factors, impacting on hepatic glucose and lipid metabolism. The following contents describe the molecular mechanisms and/or signaling pathways that are regulated by PPARγ, so as to reveal the dysregulation of lipid metabolism in hepatocytes and therefore contribution to fatty liver disease. It will be beneficial for understanding the pathogenesis of fatty liver disease in dairy cows and provide a molecular basis for the treatment and prediction of the disease. 

## 5. PPARγ Directly Regulates Lipid Metabolism in the Liver

PPARγ regulates target gene expression in adipocytes, participating in adipocyte differentiation [52,57] and regulating lipid metabolism [57,58,59], and primarily regulates signal transduction in pancreatic islet cells, greatly contributing to the occurrence and development of NAFLD. In addition to its significance in adipocyte differentiation, PPARγ is crucial in mediating lipid oxidation and lipogenesis [60,61]. Leptin-deficient obese (ob/ob) mice with inhibited liver specific PPARγ exhibit significant alleviation of fat deposition in liver, but along with aggravated hyperglycemia and IR [62,63]. Deletion of PPARγ in hepatocytes and macrophages prevent mice from developing hepatic steatosis [64,65]. The expression of PPARγ and lipogenesis-related genes, such as the fatty acid transporter *CD36*, are significantly elevated in the liver during hepatic steatosis, along with increased TAG level in the liver [66] (Figure 1). The above findings suggest that PPARγ activation promotes lipid deposition in the liver [67].

The downstream target of PPARγ, CD36, is a scavenger receptor class B that is primarily involved in the membrane transport of medium and long chain fatty acids. In macrophages, PPARγ and RXR (retinoid X receptor) form a heterodimer that binds to the specific PPARγ response element located in the promoter region of *CD36* gene and therefore increases *CD36* mRNA (messanger ribonuclei acid) level [68]. Compared to the mice fed with a normal diet, mice fed with high-fat diet after 4 weeks of administration showed significant upregulation of PPARγ2 and increased TAG content in hepatocytes. In contrast, silencing PPARγ2 using an adenoviral siRNA vector inhibits the expression of *CD36* and subsequently reduces TAG content in liver [69]. In experimental models without adipose tissues, liver PPARγ participates in lipid and glucose metabolism. However, in the presence of adipose tissues, liver PPARγ has a minimal effect on glucose metabolism in the liver [70], suggesting that PPARγ mainly regulates lipid metabolism in the liver.

The observations above indicate that PPARγ regulates the expression of the downstream target (*CD36*), thereby enabling TAG deposition and affecting lipid metabolism in the liver and plays a vital role in the occurrence of fatty liver disease (Wang et al., unpublished data; Figure 1). Our results also indicated that the downstream target genes of PPARγ were regulated by scaffold protein menin and NAD^+^ (nicotinamide adenine dinucleotide)-dependent class III histone deacetylase sirtuin 1 (SIRT1) at the transcription level, affecting lipid deposition in hepatocytes (Li et al., unpublished data).

## 6. PPARγ Indirectly Participates in Lipid Metabolism via Lipid Oxidation

Activated PPARγ regulates the expression of oxidative stress-related factors. After PPARγ ligand activation, PPAR/RXR undergoes conformational changes and associates with coactivators binding to PPARγ response elements locates in target genes, thereby regulating the transcription of these target genes [45,71,72]. Cytokines such as TNFα (tumor necrosis factor α), IFN-γ (interferon-γ), IL-1 (interleukin-1), IL-2 (interleukin-2), and IL-6 (interleukin-6) inhibit lipogenesis in adipose tissues. However, activated PPARγ can decreases cytokine levels, thereby promoting lipogenesis and causing inflammation reaction [73,74] (Figure 1). Activated PPARγ expression in macrophages is confirmed to play anti-inflammatory roles in humans and rodents [74,75]. Moreover, PPARγ ligands stimulate macrophage apoptosis [74,76,77]. Lipopolysaccharides are an important etiological factor for the systemic inflammatory response disease. PPARγ activation inhibits the activity of lipopolysaccharide and IL-1 by reducing DNA (deoxyribonucleic acid) binding and transcription by nuclear factor-κB (NFκB), activator protein-1, and signal transducer and activator of transcription 1, thereby directly inhibiting the expression of pro-inflammatory genes and indirectly promoting liver lipogenesis resulting in excessive lipid accumulation in the liver [78,79,80]. PPARγ decreases the expression of inflammatory cytokines TNFα and IL-1, and therefore suppressing the inflammation-inducing effects and oxidative stress. In contrast, these inflammation factors may inhibit lipogenesis. Reducing the level of these inflammatory factors indirectly promotes lipogenesis and stimulates lipid deposition, causing the incidence of fatty liver disease.

## 7. PPARγ Indirectly Participates in Lipid Metabolism via Insulin Resistance

Insulin resistance (IR) refers to impaired normal physiological function of insulin, therefore the target organs have reduced sensitivity to insulin. Studies have shown that IR is the main cause of NAFLD [81,82]. As a result of IR, serum fatty acid levels are increased and produce damaging reactive oxygen species (ROS). Oxidative stress may be exacerbated further by ultrastructural mitochondrial lesions, which impair respiratory chain function. Insulin inhibits β-oxidation in the mitochondria through modifying the protein acetylation [83,84,85], resulting in the accumulation of lipids in hepatocytes.

A study found that individuals with PPARγ deletions suffer from early-onset type Ⅱ diabetes accompanied with severe IR [50]. Adipocytokines, such as inflammatory factors (TNF-α and IL-6), released during obesity by adipose tissues affect host tissues and target organs (liver) and cause IR in the liver (Figure 1). Hence, PPARγ is an important candidate for IR suppression. Upon PPARγ activation by its ligand, insulin sensitivity of cells increases, promoting the expression of sterol regulatory element-binding protein-1, fatty acid-binding protein-4, and lipases in adipocytes, and increasing free fatty acid uptake and de novo synthesis of TAG in liver tissues [61,86,87].

Adiponectin (ADPN) is an endogenous bioactive protein that is secreted by adipocytes. ADPN is an insulin-sensitizing hormone that alleviates IR. PPARγ activation increases ADPN mRNA stability and promotes ADPN synthesis, stability, and release [88]. PPARγ also upregulates the expression of insulin-dependent glucose transporter 4 (GLUT4) and phosphoinositide 3-kinase (PI3K) in adipose and muscle tissues, induces the expression of casitas B-lineage lymphoma (CBLB) that participates in insulin signaling, and inhibits the expression of suppressor of cytokine signaling 3 that participates in IR to maintain insulin sensitivity in adipose tissues and skeletal muscles [65,88,89,90]. PPARγ regulates insulin signal transduction and the expression of ADPN and inflammatory proteins to modulate the degree of hepatic IR (Figure 1), playing an important regulatory role in the accumulation of TAG in the liver.

## 8. PPARγ Indirectly Participates in Lipid Metabolism via Gluconeogenesis

Gluconeogenesis is the process in which non-carbohydrate precursors are converted to glucose or glycogen. Diabetes and fatty liver disease involve gluconeogenesis and dysregulated glycogenolysis resulting in increased hepatic glucose output [91,92].

PPARγ is an important target for treating disorders involving lipid metabolism, IR, and gluconeogenesis [82,86]. A reduction in PPARγ expression in the liver increases the expression of hexokinase and reduces that of gluconeogenesis enzymes, such as phosphoenolpyruvate carboxykinase (PEPCK) and glucose-6-phosphatase (G6P), thereby inhibiting hepatic gluconeogenesis and deposition of TAG in the liver [93]. Activated PPARγ enhances the expression of the phosphoinositide 3-kinase subunit p85 and promotes hepatic gluconeogenesis (Figure 1) [94]. Activated PPARγ also increases the synthesis of glucagon, influx of amino acids into hepatocytes, and conversion to glucose via gluconeogenesis. Accordingly, PPARγ directly and indirectly regulates the expression of enzymes and proteins involved in hepatic gluconeogenesis. Thus, PPARγ is an important upstream regulator increasing the incidence of diabetes and fatty liver disease [95].

In summary, PPARγ is a transcription factor that regulates adipocyte differentiation, lipid accumulation, fatty acid oxidation and synthesis, oxidative stress, IR, and expression of the gluconeogenesis-related genes, therefore modulating the biological processes involved in hepatic lipid metabolism (Figure 1). Accordingly, PPARγ is an important protein that regulates the pathogenesis of NAFLD. Investigations on the agonists and antagonists of PPARγ might provide novel ideas for the development of drugs against NLFLD. 

## 9. Conclusions and Outlook

NAFLD is a clinico-pathologically defined process associated with metabolic syndrome [23] and fundamentally pin-pointed to the pathogenesis of lipid metabolism, causing, for example, type Ⅱ diabetes, obesity, either development of cardiovascular diseases or cirrhosis, and hepatocellular carcinoma (HCC), threatening the health of humans and animals. The disease is characterized histologically steatosis and other parenchymal changes, ranging from inflammation to hepatocyte apoptosis/necrosis to fibrosis. NAFLD has become a globally occurring chronic disease that threatens the health of both humans and animals [20,21,96]. However, there is no efficacious drug available that can directly be used to treat NAFLD. Cellular stress and immune reactions, as well as lipid metabolism, had been implicated in the pathogenesis of in animal NAFLD models [23]. There was ample evidence of the positive effects of dietary antioxidant polyphenols, carotenoids, and glucosinolates on the reversion of NAFLD [34,97], although the mechanism of their action was not yet fully elucidated. Discovery and revealing the mechanisms of the important regulatory factors/genes, such as PPARγ, that regulate the pathogenesis of this disease is pertinent and imperative for preventing and treating fatty liver disease in humans and animals. Understanding the molecular mechanisms of the pathogenesis of fatty liver disease will further enhance our understanding of NAFLD, developing safer and effective therapeutics to prevent and/or treat fatty liver disease in humans and animals.

Fatty liver syndrome is a typical type of NAFLD frequently occurring in dairy cows in the perinatal period, caused by negative nutrient balance after calving. The fatty liver disease that occurs in dairy cows is a good animal model to reveal the pathology and pathogenesis of NAFLD. More recent study suggests that impaired hepatic mitochondrial function (such as protein lysine acetylation) is closely associated with fatty liver disease during early lactation in dairy cows [43,84]. Determining the crucial genetic factors, such as PPARγ, will also provide essential clues in breeding improvement of fatty liver disease-resistant dairy cattle, eventually contributing to sustainable development of dairy industry. 

## Appendix A

**Table A1:** Biological processes associated with NAFLD are regulated by PPARγ through different signaling factors/proteins.

Biological Processes	Proteins or Signaling Factors That Interact with PPARγ		
	Protein Name	Protein Description or Abbreviation	References
Lipid Metabolism	NRF2	Nuclear factor E2-related factor 2	[45]
	CD36	Fatty acid translocase	[66]
	RXR	Retinoid X receptor	[68]
Oxidative Stress	NRF2	Nuclear factor E2-related factor 2	[45,46]
	IFN-γ	Interferon-γ	[74]
	IL-1	Interleukin-1	[74]
	IL-2	Interleukin-2	[74]
	IL-6	Interleukin-6	[74]
	LPS	Lipopolysaccharide	[78]
	NF-κB	Nuclear factor kappa B	[79]
	AP-1	Activator protein-1	[78]
	STAT-1	Signal transducers and activators of transcription 1	[78]
	TNF-α	Tumor necrosis factor α	[86]
Insulin Resistance	SREBP-1	Sterol-regulatory element binding protein-1	[86]
	TNF-α	Tumor necrosis factor α	[86]
	ADPN	Adiponectin	[88]
	CBLB	Casitas B-lineage lymphoma	[88]
	SOCS3	Suppressor of cytokine signaling 3	[88]
	AKT	Protein kinase B	[89]
	GLUT4	Glucose transport protein 4	[89]
	PI3K	Phosphoinositide 3-kinase	[89]
Endoplasmic Reticulum Stress	ATF6	Activating Transcription Factor 6	[29]
	GRP78	Glucose regulated protein 78	[30]
	IRE1α	Inositol-requiring enzyme-1α	[31]
	NRF2	Nuclear factor E2-related factor 2	[45]
	TNF-α	Tumor necrosis factor α	[86]
Gluconeogenesis	HK	Histinine kinase	[93]
	PEPCK	Phosphoenolpyruvate carboxykinase	[93]
	G6P	Glucose-6-phosphate	[93]
	PI3K	Phosphoinositide 3-kinase	[94]

NAFLD, non-alcoholic fatty liver disease; PPAR**γ,** peroxisome proliferator-activated receptor γ.
